# Hospital nurses’ attitudes, negative perceptions, and negative acts regarding workplace bullying

**DOI:** 10.1186/s12991-017-0156-0

**Published:** 2017-09-15

**Authors:** Shu-Ching Ma, Hsiu-Hung Wang, Tsair-Wei Chien

**Affiliations:** 1 College of Nursing, Kaohsiung Medical University, Kaohsiung, Taiwan; 2 Nursing Department, Chi-Mei Medical Center, Tainan, Taiwan; 3 Research Department, Chi-Mei Medical Center, 901 Chung Hwa Road, Yung Kung Dist., Tainan, 710 Taiwan; 4 Department of Hospital and Health Care Administration, Chia-Nan University of Pharmacy and Science, Tainan, Taiwan; 5 Bachelor Program of Senior Services, Southern Taiwan University of Science and Technology, Tainan, Taiwan

**Keywords:** Workplace bullying, Theory of planned behavior, Rasch measurement, Path analysis, Partial least squares structural equation modeling

## Abstract

**Background:**

Workplace bullying is a prevalent problem in today’s work places that has adverse effects on both bullying victims and organizations. To investigate the predictors of workplace bullying is an important task to prevent bullying victims of nurses in hospitals.

**Objective:**

This study aims to explore the relationships among nurses’ attitudes, negative perceptions, and negative acts regarding workplace bullying under the framework of the theory of planned behavior (TPB).

**Methods:**

A total of 811 nurses from three hospitals in Taiwan were surveyed. Nurses’ responses to the 201 items of 10 scales were calibrated using Rasch analysis and then subjected to path analysis with partial least-squares structural equation modeling (PLS-SEM).

**Results:**

The instrumental attitude was significant predictors of nurses’ negative perceptions to be bullied in the workplace. Instead, the other TPB components of subjective norm and perceived behavioral control were not effective predictors of nurses’ negative acts regarding workplace bullying.

**Conclusions:**

The findings provided hospital nurse management with important implications for prevention of bullying, particularly to them who are tasked with providing safer and more productive workplaces to hospital nurses. Awareness of workplace bullying was recommended to other kinds of workplaces for further studies in future.

**Electronic supplementary material:**

The online version of this article (doi:10.1186/s12991-017-0156-0) contains supplementary material, which is available to authorized users.

## Introduction

Workplace bullying occurs when an employee experiences a persistent pattern of mistreatment from others in the workplace that causes harm [1]. Likely, workplace bullying is persistent exposure to interpersonal aggression and mistreatment from colleagues, superiors, or subordinates [2, 3]. The form of bullying can include such expressions as verbal, nonverbal, psychological, physical abuse, humiliation and cyber [4]. Unlike the forms of school bullying in the workplace bullying, workplace bullying is in the majority of cases reported as having been perpetrated by someone in authority over the target, sometimes from peers, and occasionally from subordinates [5].

Bullying can be covert or overt. It is frequently missed by superiors and well known by many employees throughout the organization. Researchers have done impressive studies investigating this problem by determining its frequency, identifying groups at risk in different occupational groups and sectors [6], addressing prevalence of bullying in different countries and among different occupational groups [7], reporting the impact on bullying in a workplace and the group-level processes that impact on the incidence and maintenance of bullying behavior [8], detecting the appropriateness of level of the bully scaling [9], testing a multidimensional model of bullying in the nursing workplace [10], and even exploring a computer adaptive testing (CAT) tactic to examine hospital nurses’ perception of workplace bullying [11]. However, they all have focused on only one aspect of assessment regarding bully attitudes or negative acts, or merely on a single correlation between attitudes and negative acts but failed to investigate the correlation between these variables under a sound theoretical framework, for instance, using theory of planned behavior (TPB) [12] to examine the relations of those variable domains.

### PLS-SEM used for exploring the relationships between these variables

The counterproductively negative effects like bully are not limited to the targeted individuals but led to a decline in employee morale and a change in an organizational culture. None to date was to fill this gap through a comprehensively overall viewpoint by exploring the relationships among nurses’ attitudes, negative perceptions, and negative acts regarding workplace bullying under the framework of the TPB, particularly, using the method of partial least squares structural equation modeling (PLS-SEM). The PLS-SEM is evolving as a statistical modeling technique and its use has increased exponentially in recent years within a variety of disciplines, due to the recognition that PLS-SEM’s distinctive methodological features make it a viable alternative to the more popular covariance-based SEM approach [13] in social sciences.

### Theory of planned behavior applied to this study

**Fig. 1:**
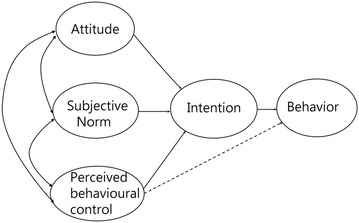
The model of theory of planned behavior

The TPB proposed by Ajzen [12, 14] is a rigorous theoretical framework to provide prediction and explanation of examinees’ intentions to behavior. TPB has been successfully applied to provide a better interpretation of diverse behaviors in western settings [15]. According to TPB theory, three determinants—including attitude (i.e., whether I want to or not to support something), subjective norms (i.e., whether others encourage or limit me to support or not to support something), and perceived behavioral control (i.e., whether I have opportunities and resources to do or not to do something)—exert their effects on behavior through intentions [12] presented in Fig. 1.

In the current study, we applied the counterproductive TPB (CP-TPB for short in this study) concept to the bully negative acts through its underlying negative perceptions predicted by the other three determinants (i.e., attitude, subjective norms, and perceived behavioral control).

Attitude is denoted as the personal orientation with a positive or negative thinking. The orientation often contains two components: (1) affective and (2) instrumental attitude [12, 16]. The affective attitude is related to feelings or emotions positively or negatively toward a target; while instrumental attitude carries an appraisal of the consequences of the target. As such, a paper [17] reported that the negative Automatic Thoughts Questionnaires (ATQ) score [18] was positively correlated with the Liebowitz Social Anxiety Scale (LSAS) scores and the positive ATQ score was negatively correlated with the LSAS scores [17].

Subjective norm refers to perceived social pressure from others to perform (or not perform) the behavior. Perceived behavioral control refers to one’s perception of the ability and control over the target. Self-efficacy is a perceived ability or a controllability, which refers to people’s beliefs that they have the ability to resist any negative acts such as the bully behavior.

Negative acts might be perceived by a series of negative feelings such as poor mental health, burnout, and intention to resign. That is, one holding a negative attitude is possible to yield negative perceptions that might lead to a feeling of all negative acts around him or her. For more information about the definition of each study scale, we provide a couple of practical examples (e.g., original questions) on their specific meaning and implication in Additional file 1.

Generally, those with a favorable attitude, positive subjective norms, and a high level of perceived behavioral control (i.e., self-efficacy) will more likely earn a low probability of negative perceptions and negative acts. Similarly, a low degree of organizational subject norm and personal perceived behavioral control possibly links to negative acts through the negative perceptions. It is required to replace the TPB from need-to-do-it with the CP-TPB avoidance-to-do-it for interpreting the relationship of components with a study model.

### Aims of the study

This study aims at examining the extent to which CP-TPB can predict and explain nurses’ negative perceptions and negative acts of a bullied victim in a hospital. The following two specific hypotheses are tested in Fig. 1:

(1) Nurses’ negative perceptions to negative acts can be predicted by attitude, subjective norm, and perceived behavioral control regarding workplace bullying; and (2) nurses’ negative acts can be predicted by negative perceptions and perceived behavioral control regarding workplace bullying.

## Methods

### Study participants

The study sample was randomly selected and recruited using the last 3 digits of the identification card number from nurses of a group of hospitals with 2133 beds in southern Taiwan in the summer of 2012. As an incentive for participation, a gift consumer card for US$6.40 good for purchases at 7–11 convenience stores was offered to participants. A total of 811 nurses completed 201 items for 10 scales (e.g., each one illustrated by several items, see Additional file 1). This study was approved and monitored by the Research Ethics Review Board of the Chi-Mei Medical Center. Demographic data collected included gender, work tenure in hospitals of all types, age, marital status, and education level.

### Instruments

A Nurse’s Conceptions Regarding Workplace Bullying Questionnaire containing 201 item of 10 scales was developed to assess the five components in the TPB framework in Fig. 1. The scales included two parts: (A) negatively inversed scores (the higher scores, the more negative perceptions or acts): workplace bullying, intent to resign, nurse burnout, and personal mental disorder, (B) positively increased monotonically scores (the higher scores, the more positive effects on persons or organizations): job satisfactory, service spirit, authority distance, leadership of nurse superiors, organization culture, and personality. Participants were asked to rate each item on a 5-point scale with response options ranging from strongly disagree (1) to strongly agree (5). A higher score represents a higher level of the respective latent trait of the aforementioned two kinds of negative and positive scales under investigation.

The scale development procedure was guided by DeVellis’ instruction [19] and item crafting was guided by Ajzen’s principles [16] for TPB scale construction. The questions were constructed based on previous literature on workplace bullying [9–11], and consultative discussions with relevant experts (in the field of health care assessment) as well as frontline nurses who have had first-hand experiences in nursing care. The scales were then subjected to a pilot test on a small sample of nurses (*n* = 32) for the purpose of helping refine those questions by identifying ambiguities and anomalies in items of wording, as well as possible bias.

The scores on negatively worded items just in Authority distance scale were reversed before the data analysis so as to maintain the consistency of interpretation. The other three scales of mental disorder, burnout, and intent to resign with negatively worded items are kept with original codes for data analysis.

### Data analysis

Two analytical methods, i.e., Rasch analysis [20] and path analysis, were used in the present study. Rasch rating scale analysis using Winsteps 3.7 [21] was used for examining the psychometric properties of the ten scales and for calibrating nurse’ (person) measures on each of the ten latent traits. The Rasch estimated person measures were subsequently analyzed by path analysis using PLS-SEM [13] to investigate the relationships among components under CP-TPB model. This approach to data analysis differs from the conventional SEM method containing all indicators to fulfill the function of measurement model. In contrast, we applied Rasch analysis to PLS-SEM for measuring the latent (unobserved) traits using those ten underlying measures.

An inherent weakness associated with conventional analytical techniques based on classic test theory (CTT), such as factor analysis, is that they require linear, interval scale data input [22]. Raw data collected through Likert-type scales are always ordinal since their categories indicate its ordering without any proportional levels of meaning [23, 24]. Therefore, it is highly possible misleading conclusions if applying CTT to raw scores which are ordinal data (i.e., response from 1 to 5 ordered category) in nature.

The Rasch model overcomes this problem by converting ordinal data into interval measures which have a constant interval meaning and provide objective measurement from ordered category responses [24]. Once the interval metric is established, person measures and item difficulties are to be calibrated onto a single unidimensional latent trait continuum which facilitates direct comparisons between person measures and item difficulties. Empirically, Rasch analysis has been successfully applied in education and social sciences in addressing assessment issues [23, 25, 26].

Multiple criteria including Rasch person/item reliability, item fit statistics, the amount of variance explained by each of the scale measures, and step thresholds are used to examine the psychometric properties of those scales. Rasch person/item reliability estimates the replicability of person/item ordering along the latent trait metric [23]. Item fit statistics estimate the extent to which the data matches the measurement specifications of the Rasch model. Outfit and infit mean squares (MNSQ) are widely used indices of item fit statistics. The values of Outfit and Infit MNSQ (range from 0 to positive infinity) with 1.0 indicating the (unattainable) perfect fit to the Rasch model. Researchers [27] suggested that MNSQs falling in the range of 0.6 and 1.4 indicated a productive measurement for survey data with rating scales. This criterion (i.e., MNSQ in a range of 0.6 and 1.4) was used as the cut-off value of MNSQ fit statistics in this study. Variance explained by Rasch measures refers to the proportion of variance in the observed data which can be explained by the Rasch measures [25]. A higher proportion of variance indicates that the Rasch model better predicts both items and persons. Step threshold difficulties are examined to ensure the appropriate category functioning of the rating scales by Linacre suggested guidelines [28].

In path analysis, the PLS-SEM method [13, 29] was applied to investigate the correlation between components under the theoretical framework of CP-TPB model. The path coefficients between any two components under the CP-TPB using PLS-SEM were examined by the criterion of type I error at 0.05 level.

## Results

### Psychometric properties of the scales

The psychometric properties of the ten scales were investigated from a Rasch measurement perspective. Any item mis-fitting to the Rasch model (both infit and outfit MNSQ being higher than 1.4) was removed from the responding scale with an approach of one at a time (i.e., each run just for one deleted item) according to the misfit order [30], and re-applied Rasch analysis until all remaining items showed sufficient fit to the Rasch model. Table 1 presents the summary of psychometric properties of all scales.

**Table 1:** Psychometric properties of measurement scales

Scale	No. of items	Rasch person/item reliability	Variance explained by measures (%)	Step threshold			
				Step 1	Step 2	Step 3	Step 4
A. The higher scores, the more negative perceptions or acts							
Bullying acts	22	0.90/0.98	41.90	−2.30	−0.13	0.44	1.99
Intent to resign	6	0.76/1.00	51.80	−1.36	−0.57	0.20	1.65
Burnout	11	0.90/1.00	61.40	−3.89	−1.00	1.76	3.13
Mental disorder	6	0.83/1.00	67.20	−4.19	−0.69	1.81	3.07
B. The higher scores, the more positive effects on persons or organizations							
Job satisfaction	20	0.92/0.99	51.30	−3.77	−2.52	0.89	5.39
Service spirit	17	0.92/1.00	56.90	−4.73	−2.63	1.49	5.78
Authority distance	6	0.75/0.99	43.90	−2.96	−0.53	0.95	2.54
Leadership	50	0.83/1.00	42.50	−2.30	−0.48	0.50	2.29
Organization culture	20	0.84/0.97	41.00	−2.32	−1.00	0.42	2.00
Personality	43	0.91/1.00	44.50	−2.97	−1.39	0.56	3.81

It is shown in Table 1 that the Rasch person/item reliabilities for all scales are higher than 0.80 except the person reliability for the intent to resign and authority distance scales due to a short length of items. Rasch measures explained over 40% of the variables observed in the data for all scales. The results indicated that all the ten scales had quite good psychometric properties at an acceptable scaling quality. Table 1 presents the summary of psychometric properties of all scales. The final sample items of the scales are illustrated in Additional file 1.

The category functioning of the rating scales were examined to determine whether respondents used all response opportunities appropriately. It can be seen that the step thresholds (the intersection point between consecutive categories) advanced monotonically with the category, indicating that the 5-point rating scale functioned rather well, and meaning higher performance categories corresponded to higher measures of the latent trait. In summary, the results showed that the scales were psychometrically robust enough for use with the sample in the current study.

### Descriptive statistics

Descriptive statistics were undertaken to provide an overall viewpoint of the interval Rasch-calibrated measures of nurses on the ten constructs. Table 2 presents means (in a log odds unit) and standard deviations (SD) of nurses’ measures on the scales as well as Pearson correlations among the constructs of interest. In Rasch analysis, the mean of item difficulties is arbitrarily set to zero and the interpretation of item difficulties and person measures are based on pair-wise comparisons between items and persons. Therefore, person measures higher than zero indicate a positive response, while person measures lower than zero that indicate a negative response (e.g., the first four scales with negatively inversed scores present not serious because scores are less than zero).

**Table 2:** Means, standard deviations, and correlations of the study components

	Component	Mean	SD	(1)	(2)	(3)	(4)	(5)	(6)	(7)	(8)	(9)
(1)	Bullying acts	−4.76	1.84	–								
(2)	Intent to resign	−0.76	0.89	0.23**	–							
(3)	Burnout	−1.64	2.08	0.26**	0.23**	–						
(4)	Mental disorder	−3.54	2.64	0.34**	0.14**	0.47**	–					
(5)	Job satisfaction	1.36	1.88	−0.32**	−0.29**	−0.39**	−0.3**	–				
(6)	Service spirit	3.13	2.20	−0.18**	−0.19**	−0.25**	−0.12**	0.61**	–			
(7)	Authority distance	1.31	1.26	0.02	0.01	0.03	0.00	−0.06	−0.06	–		
(8)	Leadership	1.55	0.49	−0.02	−0.02	0.00	0.04	−0.04	0.00	0.22**	–	
(9)	Organization culture	0.70	1.19	−0.02	−0.01	0.01	0.03	0.02	0.00	−0.07*	0.22**	–
(10)	Personality	2.76	0.92	−0.04	−0.04	−0.01	0.01	0.02	0.02	−0.02	0.10	0.33**

* *p* < 0.05; ** *p* < 0.01

It is shown in Table 2 that, in general, nurses held a substantially low level of negative perceptions [i.e., scales (2)–(4)] to potentially be bullied victims [i.e., the scale (1)] with means less than zero. Attitude, both job satisfaction (mean = 1.36) and service spirit (=3.13), and subject norm with means of 1.31 and 1.55 regarding authority distance and leadership were quite positive, while perceived behavioral controls, i.e., organization culture (mean = 0.70) and personality (mean = 2.76), were positive.

It is worth noting that all of those respective latent traits were significantly related within the component (i.e., with a good convergent validity) and unrelated between components (i.e., with a good discriminant validity). Nurses had a slightly negative mean measure on bully acts (mean = −4.76). This indicates that, according to the direct effect from the nearby nurses’ negative perceptions and the indirect effect from the three far-left components, a rather good hospital climate was evident and led to a lower negative perceptions and a very low negative acts of workplace bullying.

### Results of path analysis

Nurses Rasch-calibrated measures on the CP-TPB components are subsequently subjected to path analysis, aiming at addressing the main research questions: to explore whether nurses’ negative perceptions to negative acts can be predicted by attitude, subjective norm, and perceived behavioral control regarding workplace bullying. The results of path analysis showed that the standardized regression weight of the path (−0.40) from attitude to negative perceptions was significant (*p* < 0.01). The paths from subjective norm (=−0.006) and perceived behavioral control (=0.004) to negative perceptions were not significant. The correlations between the three far-left components were virtually zero. The standardized regression weight of the path from negative perceptions to negative acts (=0.38) was significant (*p* < 0.01), while the paths from perceived behavioral control component (=−0.03) were not significant.

**Fig. 2:**
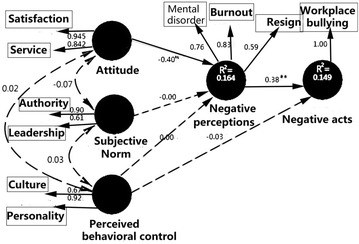
Path analysis based on the CP-TPB (**p < 0.01)

The proposed model accounts for 16.4% of the variances in nurses’ negative perceptions with a statistical power of 0.99 (calculated by 3 predictors, 813 sample size, probability level at 0.05, and observed *R* ^2^ = 0.164) and accounts for 14.9% of the variances with a statistical power of 0.99 (computed by 2 predictors, 813 sample size, probability level at 0.05, and observed *R* ^2^ = 0.149) in nurses’ negative acts. The relationships among the latent traits in the CP-TPB are presented in Fig. 2.

The direct and indirect effect coefficients of predictors on workplace bullying are presented in Table 3. In the proposed model, negative perceptions (via direct effect) had a substantial positive effect (=0.38) on negative acts; attitude predictor (via indirect effect) had a moderate negative effect (=−0.16) on negative acts, while the other predictors had a far less than substantial effect on negative acts.

**Table 3:** Effects of each latent variable on the bully behavior

Predictor components	Direct effect	Indirect effect	Total effect
Negative perceptions	0.38	–	0.38
Attitude	–	−0.16	−0.16
Subjective norm	–	−0.01	−0.01
Perceived behavioral control	−0.04	–	−0.04

## Discussion

This study used CP-TPB as a theoretical framework to verify that (1) nurses’ negative perceptions to negative acts can be predicted by personal attitude; and (2) nurses’ negative acts regarding workplace bullying can be predicted by negative perceptions.

### What this adds to what was known

Many previous pieces of research [5–11] have investigated the impact on bullying in a workplace and the group-level processes that impact on the incidence and maintenance of bullying behavior, but few attempts have been made to build a structural understanding of workplace bullying or of the relationships among variables which have influences on nurses’ negative perceptions and negative acts of bullies.

The approach adopted for the analysis was a two-step process with Rasch analysis followed by path analysis. Rasch model can convert ordinal data into interval measures [24] and deal with missing data [31, 32] which are problematic in CTT approaches [33–35]. The Rasch person reliabilities for the intent to resign and authority distance scales are less than 0.80 in Table 1. It is because a shorter length of six items.

In path analysis, the PLS-SEM method has recently gained increasing attention, especially for the management information systems [36], as well as in marketing [37] and strategic management [38] disciplines, but also in accounting [39], family business research [40], operations management [41], and in organizational research [42]. The method is currently regarded as suitable and, to some extent, a favorable alternative to the more restrictive traditionally used covariance-based SEM (CB-SEM) method [43].

Among nurses, the prevalence of bullying was reported to be widespread [44, 45], with estimates suggesting 80% of nurses experience bullying at some point in their working lives [46]. The consequences of bullying include the following: severe psychological trauma [47]; lowered self-esteem [48]; depression and anxiety [49]; post-traumatic stress disorder [50]; physical illness [51]; financial loss; and the eventual inability to work [52]. The ripple effect of bullying also extends to family members who are liable to experience considerable stress from living with a family member who has been bullied [53]. The findings of this study can provide hospital nurse management with important implications for prevention of bullying, particularly to them who are tasked with providing safer and more productive workplaces to hospital nurses.

### What it implies and what should be changed?

The explanatory model of bullying resulting from this study in Fig. 2 identifies three components (low attitude → negative perceptions → negative acts) relations that contribute to bullying features, the relationship between bullying acts and the antecedent predictors. The model provides an insight into developing additional strategies to manage workplace bullying. In particular, the model may assist nurse managers to understand features of the work climate that perpetuate the bully behavior. Importantly, the model draws attention to personal attitude (i.e., satisfying nurse job and enjoying healthcare service) that contribute to reduce personal negative perceptions and negative acts regarding workplace bullying.

### Strengths of this study

We applied Rasch model to detect data unidimensional, to establish interval metric for SEM modeling, and to interpret the relationship between components of latent traits. Empirically, Rasch analysis has been successfully applied in education and social sciences in addressing assessment issues [23, 25, 26] and worth applying to this study.

The Rasch estimated person measures were subsequently analyzed by path analysis using PLS-SEM [13] to investigate the relationships among components under CP-TPB model. The approach differs from the conventional SEM method containing all indicators to fulfill the function of the measurement model. We, on the other hands, applied Rasch analysis to PLS-SEM for measuring the latent (unobserved) traits using those ten underlying measures.

Structural equation modeling (SEM) has become the methodology of choice for many social science researchers investigating complex relationships between latent constructs, such as those ten components in this study. Compared with another commonly used approach of CB-SEM subjected to data normal distribution assumptions and to have a not-too-small sample size, there are many advantages in applying PLS-SEM [43].

### Definitions of being bullied and engaging in bullying

This study sample was drawn from those frontline nurses who have had first-hand experiences in nursing care. All questions of the 22-item Negative Acts Questionnaire-Revised (NAQ-R) questionnaire [54] are pertaining to frequency of being bullied. We adopted the definition of bullying [55]: *when a person is teased repeatedly in a way he or she does not like… But it is not bullying when two students of about the same strength or power argue or fight. It is also not bullying when a student is teased in a friendly and playful way*.

Olweus [56] identified three prominent characteristics of bullying behavior: negative actions, repetition, and power imbalance. The negative perceptions were thus rated by victims of bullying instead of those nurses who engage in bullying behavior. Otherwise, those scale quality indices shown in Table 1 and Fig. 2 will be explicitly distorted and invalidated if nurses were confused in rating questions based on a distinct perspective of being bullied or engaging in bullying.

### Limitations and future study

The interpretation and generalization of the conclusions of this study should be carried out with caution. First, the data of this study were collected in the context of a single hospital group in Taiwan. It is worth noting that any attempt to generalize the findings of this study, especially in the prediction of workplace bullying, should be made in healthcare systems with similar social and cultural contexts.

Second, although the participants were randomly and carefully selected in a unique hospital group to represent as much different as characteristics of samples, the generalization is not as strong as that sampling from a variety of hospital groups.

Third, nurses’ perceptions were investigated by self-report data with response to such more 201 items at one moment. We cannot guarantee that all of them endorsed questionnaire with carefulness and without any cheating or guessing response.

Fourth, the 201 items of the study ten components were not included in the paper due to the space limitation. Interested readers are welcome to request the questionnaire if necessary.

The bully issue is a global problem in service-originated societies, especially among nurses in the healthcare setting [57, 58]. Our findings that nurses’ negative perceptions can be predicted by attitude as an indirect effect to negative acts are required to further prove and to induce other researches in future. For instance, in a therapeutic or care process, patients with schizophrenia, caregivers practice body restraint for protecting the individual or the community, and to facilitate transportation to health facilities might be bullying behaviors if no compassion and love exist in healthcare.

Reviewing Olweus [54] identification of bullying behaviors with three prominent characteristics: negative actions, repetition, and power imbalance. It is interesting to hypothesize that when patients with schizophrenia were treated with body restraint (negative actions) frequently (repetition) by nurse authority to patients (power imbalance), giving patient family members hold negative attitudes (or thoughts) toward nurse treatments. Comorbid mental disorders and negative perceptions will be caused, such as depression and substance drug abuse. Patient restraint will be considered as a kind of bullying behavior (i.e. negative acts).

## Conclusions

This study contributes to the academic literature by applying both Rasch analysis and PLS-SEM to explore the relationships among nurses’ attitudes, negative perceptions, and negative acts regarding workplace bullying under the framework of the TPB, which provides hospital nurse management with important implications for prevention of bullying, particularly to them who are tasked with providing safer and more productive workplaces to hospital nurses. Awareness of workplace bullying was recommended to other kinds of workplaces for further studies in future. Researchers and nurse superintendents should develop prevention and intervention programs directed at workplace bullying based on the perceived severity rather than only on the prevalence and frequency of bullying behaviors.

## Additional file

**Additional file 1.** Questions illustrated for the 10 study scales.

## Abbreviations

ATQ: Automatic Thoughts Questionnaires
CP-TPB: counterproductively negative effects on theory of planned behavior
CTT: classic test theory
LSAS: Liebowitz Social Anxiety Scale
SAD: social anxiety disorder
SD: standard deviations
TPB: theory of planned behavior
