# Supplementary material for: Hospital nurses’ attitudes, negative perceptions, and negative acts regarding workplace bullying
Source: Ann Gen Psychiatry. 2017 Sep 15;16:33. doi: 10.1186/s12991-017-0156-0 (PMC5603093; doi:10.1186/s12991-017-0156-0)
Supplement: Supplementary file 1 — Additional file 1. Questions illustrated for the 10 study scales. [file 12991_2017_156_MOESM1_ESM.docx]

**Appendix S1**: Nurse' Conceptions and Perceptions of Workplace bullying Questionnair

*represents inverse scale

1. Perceived Behavioral control: Personality(From most not adherence coded as 1 to most adherence coded as 5)

Q1. Cooperation with others rather well

Q2.Respect to others

Q3.Like to make friends

Q4 have creative ability

1. Subjective norm:*Authority distance(From most not disagree coded as 1 to most agree as 5)

Q1.My boss would not consult opinions with us when making a key decision.

Q2.My boss seldom hears subordinators' opinions.

Q3.Subordinators cannot question any to boss's decision.

1. Subjective norm: Leadership(From most not adherence coded as 1 to most adherence as 5)

Q1.My boss feels little interest to others

Q2.My boss is easily depressed and giving up for my jobs

Q3.My boss like to talk somethings that are others like to hear

1. Perceived Behavioral control: Organization culture(From most not disagree coded as 1 to most agree coded as 5)

Q1.I feel much about encouragement to think in my section

Q2.My colleagues are with high morale for working in my section

Q3.That is a section likely focussing on performance on jobs

1. Negative acts: Bully(never, occasionally, monthly, weekly, daily coded from 1 to 5)

Q1.Pressure not to claim something to which by right you are entitled

Q2.Hints or signals from others that you should quit your job

Q3.Threats of violence or physical abuse or actual abuse

1. Negative perception:*Mental disorder (never, mild, moderate, severe, very severe)

Q1.I feel sleep disorder

Q2.I feel with anxiety and nervious

Q3.I feel blue and depressive

1. Negative perception: *Intent to resign(From most not disagree coded as 1 to most agree as 5)

Q1.I want to go to other's nursing unit for work

Q2.I want to resign to other hospital for work

Q3.I want to go to other's unit without my nurse career

1. Nurse attitude: (1) Job satisfaction(From most not disagree coded as 1 to most agree coded as 5)

Q1.I am satisfied with my work loading

Q2.I feel my work promising and hopeful

Q3.I am satisfied with the job stabibility

1. Negative perception: *Burnout(Never, rare, sometimes, often, always)

Q1.I feel frustrated with my job

Q2.I feel it is hard to interact with patients

Q3.Taking care of patients lets me tired

1. Nurse attitude: Service spirit

Q1.I like to participate in activities that can upgrade hospital's image

Q2.I will actively cooprate with coleagues to achieve goals

Q3.I will paricularly notice my bahaviors and words to others
